# Current Challenges in Elucidating Respiratory Supercomplexes in Mitochondria: Methodological Obstacles

**DOI:** 10.3389/fphys.2018.00238

**Published:** 2018-03-16

**Authors:** Sehwan Jang, Sabzali Javadov

**Affiliations:** Department of Physiology, School of Medicine, University of Puerto Rico, San Juan, Puerto Rico

**Keywords:** mitochondria, ETC complexes, respiratory supercomplexes, blue native gel electrophoresis, cryo-electron microscopy, protein–protein interaction, respirasome

Mitochondrial electron transport chain (ETC) transfers electrons from NADH and FADH_2_ through complexes I–IV to oxygen, which is coupled to ATP production by the F_1_F_o_-ATP synthase (complex V). Since 2000, growing number of structural biology and biochemical studies provided strong evidence that ETC individual complexes I–IV can assemble into supramolecular structures known as supercomplexes (SCs) (Schägger, 2001). First evidence for the existence of SCs came from studies that analyzed mitochondrial membrane proteins by blue native polyacrylamide gel electrophoresis (BN-PAGE) (Schägger and Pfeiffer, 2000). Studies based on cryo-electron microscopy (cryo-EM) and refinement technology provided further insight into the structural organization of SCs at near-atomic resolution (Gu et al., 2016; Letts et al., 2016; Sousa et al., 2016). It has been suggested that supramolecular organization of SCs could enhance the catalytic activity of ETC individual complexes to transfer electrons with high efficiency through substrate channeling. The latter may reduce electron leakage and thus, prevent the production of reactive oxygen species (ROS) in mitochondria (Maranzana et al., 2013). Also, SCs could stabilize the integrity and assembly of ETC individual complexes, regulate the ETC activity and prevent aggregation of proteins in the inner mitochondrial membrane (IMM). Currently, three models known as “fluid model” (ETC complexes do not interact), “solid model” (ETC complexes are assembled in SCs), and “plasticity model” (hybrid of fluid and solid model) are commonly discussed to explain structural organization of ETC complexes (Milenkovic et al., 2017). Notably, according to BN-PAGE analysis, more than 80% of total complex I, 65% of complex III and 15% of complex IV were found in SCs in bovine heart mitochondria (Schägger and Pfeiffer, 2000).

However, despite intensive studies, mechanisms of assembling, structural organization and physiological role of SCs remain to be elucidated. Biochemical and electron microscopy studies revealed the main SC, respirasome, which contains complexes I, III, and IV (I_1_III_2_IV_1_) (Schägger and Pfeiffer, 2000; Acín-Pérez et al., 2008; Gu et al., 2016; Letts et al., 2016). According to structural biology studies, NDUFA11 and NDUFB4 subunits of complex I directly interact with the complex III subunit UQCRQ whereas the NDUFB9 and NDUFB4 subunits bind to complex III through UQCRC1 and UQCRFS1 subunits. Interestingly, complex IV is less tightly bound to complexes I and III, and the interactions of complex IV can vary in different respirasomes. A close association exists between COX7C subunit of complex IV and ND5 subunit of complex I. Also, COX7A interact with complex III subunits UQCR11, UQCRC1 and UQCRB (Letts et al., 2016; Wu et al., 2016).

In addition to respirasome, several other SCs containing the ETC complexes in various stoichiometries have been identified by both approaches. Complex II is the only one which was not associated with SCs based on BNGE (Schägger and Pfeiffer, 2000) and cryo-EM (Gu et al., 2016; Letts et al., 2016; Sousa et al., 2016) studies. Most recent cryo-EM studies including biological and structural analyses of SCs suggested the possible involvement of the complex II in respirasome (SC I_2_III_2_IV_2_) to form the megacomplex I_2_II_2_III_2_IV_2_ (Guo et al., 2017). Although existing approaches are not able to identify the complex II in SCs, a potential site of complex II can be seen in the 3D structure of the megacomplex and even in the respirasome. In favor of this, using crosslinking mass spectrometry studies reported that SDHF4, a complex II assembly factor might interact with Cox41 from complex IV (Schweppe et al., 2017). The proposed structure of the SC, which includes all four ETC complexes, could provide highly effective transfer of electrons.

Studies elucidating interactions between ETC individual complexes and mechanisms of SC assembling raises the question of whether all four ETC complexes are involved in SCs (Figure 1). Current structural biology and biochemical techniques have several limitations that do not allow researchers to answer this question. Here, we discuss challenges associated with the processing of mitochondrial samples for analysis of the natural structure of SCs. Current biochemical and structural studies of SCs by BNGE and cryo-EM are mostly based on the analysis of digitonin-solubilized mitochondrial membrane proteins. It should be noted that the structural integrity of SCs hardly depends on the concentration of digitonin and other detergents used for protein solubilization; the detergents can dissociate weakly bound complexes at high concentration whereas low concentration they may not be effective enough to solubilize all SCs. Hence, extraction of SCs is sensitive to the digitonin/protein ratio, and variabilities in the ratio have a significant effect on the qualitative and quantitative characterization of SCs (Pérez-Pérez et al., 2016).

**Figure 1:**
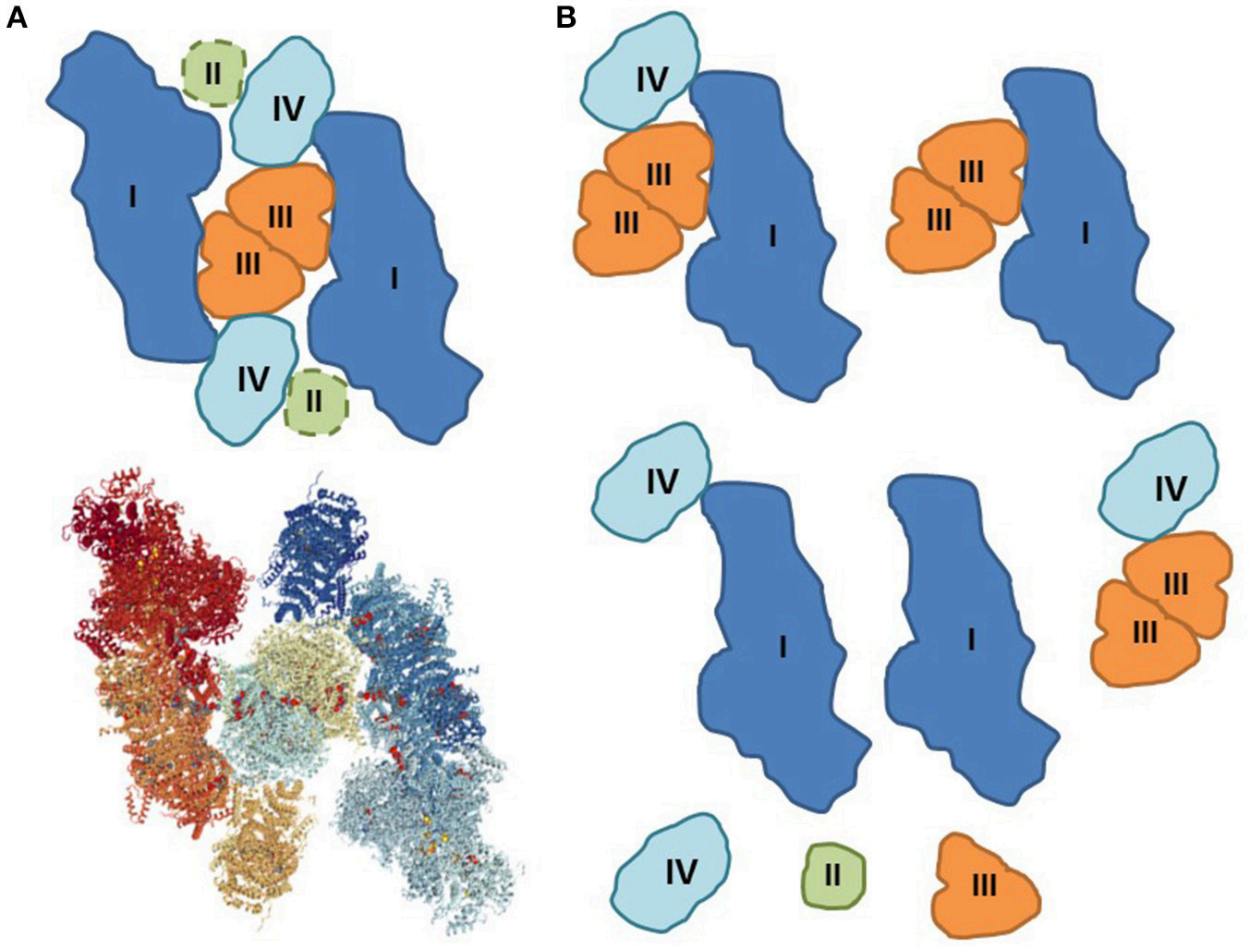
Assembling and disintegration of respiratory SCs. **(A)** Proposed structure of the megacomplex I_2_II_2_III_2_IV_2_ that can include all ETC individual complexes (Guo et al., 2017). The potential site of complex II is added as dashed lines (top). Solved 3D structure of megacomplex I_2_III_2_IV_2_ by cryo-EM (bottom) (PDB Accession: 5XTI). **(B)** The natural integrity of respiratory megacomplexes can be partially compromised during sample preparation for BNGE and cryo-EM. The disintegration of the original structures can be induced by artifacts including, among others, lack of natural environment, loss of cardiolipin, mitochondrial isolation (centrifugation and washing), solubilization (digitonin), air-water interface, and random collision.

Solubilization of membrane proteins including the SCs by detergents apparently affects their native structure. During solubilization, the integrity of solubilized particles is limited by the ability of the detergent to retain membrane-bound protein complex of higher-order structures in the aqueous solution. Hence, resulting structures are more likely to be only a part of the original structure due to partial dissociation of SCs during solubilization. Currently, only digitonin demonstrates the ability to maintain the integrity of respirasome (I_1_III_2_IV_1_). Recently, newly developed non-ionic mild detergent PCC-aM (trans-4-(trans-4′-propylcyclohexyl) cyclo-hexyl-α-D-maltoside) showed the ability to retain respirasome (Sousa et al., 2016). Digitonin aggregates readily in solution on ice, which makes it hard to maintain reproducible solubilization for multiple batches of analysis. Also, weak interactions in SC assemble could be lost due to the exposure of protein samples to the water-air interface. Structure of protein assembly at the water-air interface changes by varying solution protein concentration, ionic strength, and redox state (Liao et al., 2011).

Lack of lipid environment, particularly, cardiolipin, is another factor that can facilitate dissociation of loosely associated complexes upon solubilization of mitochondrial proteins. Cardiolipin, a unique phospholipid almost exclusively localized in the IMM, has been shown to stabilize the structural integrity of ETC individual complexes and SCs (Mileykovskaya and Dowhan, 2009; Böttinger et al., 2012). Downregulation of tafazzin, a cardiolipin-remodeling enzyme, was associated with dissociation of respirasome (McKenzie et al., 2006). Loss of cardiolipin during the solubilization process can change the natural structure of SC assembly.

Importantly, in addition to the solubilization issue, the procedure of mitochondria isolation and purification that include several steps of centrifugation and washing that could have a considerable effect on the SCs integrity. The artifacts associated with mitochondrial isolation such as changes in mitochondrial morphology (dimensions) can change cristae shape which determines the assembly and stability of SCs (Cogliati et al., 2013). The negative effects of isolation artifacts would be more significant for weak interactions that participate in SC assembling and can be easily dissociating during the purification of mitochondria.

The artifacts resulted from the isolation of mitochondria and solubilization of SCs compromise understanding the role of SCs in the pathogenesis of human diseases. Several studies revealed an association between SC disintegration and diseases. Likewise, reduced levels of ETC SCs were found in Barth syndrome patients (McKenzie et al., 2006) and tafazzin knockdown mice (Jang et al., 2017). Animal models of post-myocardial infarction heart failure (Rosca et al., 2008) and ischemia-reperfusion (Jang et al., 2017) induced dissociation of the respirasome in cardiac mitochondria. The disintegration of SCs in response to oxidative stress was dependent on the mitochondrial permeability transition inhibition of which abolished SC dissociation in cardiac mitochondria (Jang and Javadov, 2017). Since the opening of permeability transition pores stimulates excessive matrix swelling and results in cell death, these studies suggest a causative link between IMM topography and the structural integrity of SCs. Indeed, selective disruption of SCs has been proposed as a new strategy to block breast cancer (Rohlenova et al., 2017). Aging was associated with alterations of SCs in mitochondria of the brain (Frenzel et al., 2010), heart (Gómez et al., 2009) and skeletal muscle (Lombardi et al., 2009). Conversely, exercise enhanced expression of individual ETC complexes and SC formation in human skeletal muscle (Greggio et al., 2017).

However, it should be noted that SCs were analyzed in solubilized mitochondrial membranes separated by BN-PAGE in all these studies. Hence, the artifacts associated with isolation and solubilization of mitochondria, in addition to challenges in quantifying of SCs could affect the relationship between SCs assembly and functional/metabolic status of organs/tissue. For instance, 77% reduction in post-ischemic recovery of cardiac function was associated with only a 3% reduction of SCs in heart mitochondria (Jang et al., 2017).

In addition, quantification of SC bands separated by BN-PAGE is quite difficult due to overlapping of proteins. The second dimension of two-dimensional gel electrophoresis (2D-PAGE) followed by immunoblotting analysis of SCs after BN-PAGE revealed that the respirasome bands (0.9–1.0 MDa) were overlapped by the complex V dimer band (Jang et al., 2017). These bands only can be separated by linear gradient gel and over-running of the gels. Higher molecular weight (~1.2 MDa) short smear containing the complexes I, III, and IV, a part of respirasome (shown as megacomplex in Guo et al., 2017) were found to overlap with predicted complex V tetramers and heptamers (Wittig and Schägger, 2008) on the BN-PAGE gel. These bands were appeared as a short smear rather than a clear band, and cannot be separated by BN-PAGE. We attempted to quantify only respirasome signals from these bands by two-dimensional gel electrophoresis (2D-PAGE) and immunoblotting, but the results between samples were not consistent since each lane underwent series of separate procedures (equilibration, loading, running, transfer, blotting). Notably, complexes on blue native gels were highly susceptible to diffusion during equilibration before loading onto the second dimension of 2D-PAGE (Jang and Javadov, 2017; Jang et al., 2017). Also, in BN-PAGE, the size information is only approximate because of the native conformation of proteins and surface charges, which complicate the prediction of the molar mass of the identified bands. For example, respirasome (I_1_III_2_IV_1_) bands appear at 0.9–1.0 MDa on BN-PAGE gel, which predicted molar mass is 1.8 MDa. Likewise, bands of complex V dimer and complex I are seen at a much lower molar mass (0.8-0.9 MDa and 0.7-0.8 MDa, respectively) than predicted (1.4 MDa and 0.9 MDa, respectively).

Individual ETC complexes are assembled into SCs by strong and weak protein–protein interactions. The structure of the most tightly bound assemblies, like respirasome, is stabilized by phospholipids, mainly cardiolipin, and other physicochemical factors in the IMM. Although some potential assembly proteins (e.g., complex II or SDH) are a legitimate binder, they are likely to be weaker. Therefore, it is nearly impossible to precisely identify all proteins involved in SCs in isolated mitochondria *in vitro* due to quick dilution-induced dissociation by mass action. Due to abovementioned challenges and methodological obstacles, analysis of SCs *in vivo* in intact (not solubilized) biological specimens or cells (live or fixed) would be most appropriate and informative and provide direct evidence on the role of SCs in mitochondrial bioenergetics in physiological and pathological conditions.

There are a few studies that did not use detergents for solubilization of SCs. Crosslinking mass spectrometry revealed many *in situ* interactions in proteins throughout the ETC complexes, ATP synthase, and the mitochondrial contact site and cristae organizing system (Schweppe et al., 2017). Recently, Lys-Lys crosslink mass spectrometry was used to elucidate protein–protein interactions in cardiac tissue without isolation of mitochondria that supported the presence of ETC SCs assembly (Chavez et al., 2018). The study also supported previous studies on the existence of higher-order SC assembly, a megacomplex consisting of two copies each of complexes I, III, and IV (Guo et al., 2017). Likewise, disuccinimidyl sulfoxide (DSSO) crosslink mass spectroscopy of isolated mitochondria revealed that all complexes (including II and V, which have not been found so far using cryo-EM) exist in close spatial proximity thereby providing direct evidence for SC assembly in intact mitochondria (Liu et al., 2018). These crosslink techniques cannot be considered to be non-invasive but their advantage over other approaches is that they can use an intact tissue or mitochondria without solubilization, so the results may reflect natural structure and organization of SCs more precisely than the techniques using detergent-solubilized specimens. Also, they can provide valuable evidence of SC assembly free from the possibility of *in vitro* aggregation artifact.

Proximity-dependent labeling is a novel approach that can identify proximity of potentially interacting proteins and their subcellular spatial localization *in vivo* and thus, overcome technical challenges associated with detection of transient (weak) protein–protein interactions in eukaryotic cells (reviewed in Roux, 2013; Rees et al., 2015; Varnaite and MacNeill, 2016). The main principle of the technique relies on the proximity-dependent labeling of a protein of interest by the modifying enzymes that allow it to attach a tag on proximal interacting protein(s) involved in the interactome network. Subsequent purification and identification of the complexes by MS determine the interaction between proteins. The principle is used for several techniques such as proteomic proximity labeling by ascorbate peroxidase (APEX) and proximity-dependent biotin identification (BioID). APEX is capable of generating biotin-phenolxyl radicals in living cells that covalently binds to amino acids (Tyr, Trp, Cys, His) and thus, labeling proteins. This approach allows identification of subcellular localization as well as the interaction of a protein of interest with other protein(s) in cells *in vivo* (Rhee et al., 2013). The BioID technique has significant advantages over other techniques since it allows identifying the dynamic and transient protein–protein interactions *in vivo* when proteins interact indirectly or weakly with the protein of interest at proximity. Also, the technique can be applied to insoluble and inaccessible subcellular structures (Varnaite and MacNeill, 2016).

Recently, the GFP-FRET technique was used for the recording of respirasome formation in live cells. Rieger et al. determined possible colocalization of complexes III and IV in SCs by Förster resonance energy transfer (FRET) in live cells (Rieger et al., 2017). The authors implemented fluorescence lifetime imaging microscopy (FLIM) by using fluorescent sensor proteins to assess SC assembly and monitor the plasticity of SCs in live cells.

In summary, more reliable and precise techniques should be applied to overcome the existing methodological challenges that obstacle qualitative and quantitative analysis of respiratory SCs. Advanced structural biology, single-molecule labeling/imaging and biochemical techniques *in vivo* in intact live/fixed cells or tissue samples can be useful for elucidating the dynamic structure of respiratory SCs and mechanisms of their assembling/disintegration under physiological and pathological conditions.

## Author contributions

SeJ prepared a draft of the paper. SaJ critically revised the paper. Both authors reviewed and approved the final version of the paper.

### Conflict of interest statement

The authors declare that the research was conducted in the absence of any commercial or financial relationships that could be construed as a potential conflict of interest. The reviewer DS and handling Editor declared their shared affiliation.

## Abbreviations

BN-PAGE: blue native polyacrylamide gel electrophoresis
cryo-EM: cryo-electron microscopy
2D-PAGE: two-dimensional gel electrophoresis
ETC: electron transfer chain
IMM: inner mitochondrial membrane
ROS: reactive oxygen species
SC: supercomplex.
